# The inhibition of UBC13 expression and blockage of the DNMT1-CHFR-Aurora A pathway contribute to paclitaxel resistance in ovarian cancer

**DOI:** 10.1038/s41419-017-0137-x

**Published:** 2018-01-24

**Authors:** Xu Zhang, Yan Feng, Xin-Yu Wang, Ya-Nan Zhang, Chun-Nv Yuan, Song-Fa Zhang, Yuan-Ming Shen, Yun-Feng Fu, Cai-Yun Zhou, Xiao Li, Xiao-Dong Cheng, Wei-Guo Lu, Xing Xie

**Affiliations:** 1 Women’s Reproductive Health Laboratory of Zhejiang Province, Women’s Hospital, School of Medicine, Zhejiang University, Hangzhou, 310006 Zhejiang China; 2 Department of Gynecologic Oncology, Women’s Hospital, School of Medicine, Zhejiang University, Hangzhou, 310006 Zhejiang China; 3 Department of Pathology, Women’s Hospital, School of Medicine, Zhejiang University, Hangzhou, 310006 Zhejiang China

## Abstract

Paclitaxel is widely used as a first-line chemotherapeutic drug for patients with ovarian cancer and other solid cancers, but drug resistance occurs frequently, resulting in ovarian cancer still presenting as the highest lethality among all gynecological tumors. Here, using DIGE quantitative proteomics, we identified UBC13 as down-regulated in paclitaxel-resistant ovarian cancer cells, and it was further revealed by immunohistochemical staining that UBC13 low-expression was associated with poorer prognosis and shorter survival of the patients. Through gene function experiments, we found that paclitaxel exposure induced UBC13 down-regulation, and the enforced change in UBC13 expression altered the sensitivity to paclitaxel. Meanwhile, the reduction of UBC13 increased DNMT1 levels by attenuating its ubiquitination, and the up-regulated DNMT1 enhanced the CHFR promoter DNA methylation levels, leading to a reduction of CHFR expression, and an increased in the levels of Aurora A. Our findings revealed a novel function for UBC13 in regulating paclitaxel sensitivity through a DNMT1-CHFR-Aurora A pathway in ovarian cancer cells. UBC13 could potentially be employed as a therapeutic molecular drug for reversing paclitaxel resistance in ovarian cancer patients.

## Introduction

Ovarian cancer still presents the highest lethality of all gynecological tumors after decades of research, with an overall 5-year survival rate of 46%^1^. Primary cytoreductive surgery followed by combined paclitaxel and carboplatin chemotherapy is recognized as the first-line treatment strategy. In this strategy, chemotherapy is an indispensable element because surgery can not completely remove all tumor tissues, especially in advanced ovarian cancer. Although more than 80% of patients initially respond to standard chemotherapy, most of them relapse and require further therapy. Unfortunately, almost all of recurrent ovarian cancers are chemoresistant and the disease persistently progresses. Chemoresistance remains the critical cause for treatment failure and death in ovarian cancer patients. Paclitaxel, as a first-line antineoplastic agent for ovarian cancer, is used for a wide range of solid tumors, but the overall response rate is only 20–40%^2–5^. Paclitaxel resistance remains an unresolved issue although some mechanisms have been uncovered.

Ubiquitination widely exists in diverse cellular processes, such as protein degradation, the cell cycle, and signaling transduction^6,7^. Recent findings reveal that ubiquitination functions in regulating the sensitivity of tumor cells to chemotherapy agents^8–10^, including paclitaxel^8^. Yet, most of these studies focus on the relationship between chemotherapy and ubiquitin-ligases (E3), which are numerously encoded by the human genome^11^. We used a DIGE (two-dimensional fluorescence difference in gel electrophoresis) quantitative proteomic analysis to search for differentially expressed proteins between the ovarian cancer cell line SKOV3 and SKOV3-TR30, a cell line with a 27-fold increase in paclitaxel resistance over its parental SKOV3^12^, and found a remarkably decreased expression of UBC13 (UBE2N, ubiquitin conjugating enzyme E2 N) in SKOV3-TR30 cells. UBC13 is one in the family of ubiquitin-conjugating enzymes (E2) and plays a central role in ubiquitin-mediated protein degradation and signaling transduction^6,7,13^. Over the past decade, UBC13 has also been reported to be closely related to the initiation or development of various cancers^14–17^. Moreover, UBC13 was also found to mediate noncanonical ubiquitination and regulate DNA damage repair^18–20^, and to be associated with chemoresistance^21–23^. However, the relationship between UBC13 and paclitaxel is still unclear. Thus, there may be a link between UBC13 down-regulation, protein ubiquitination, and paclitaxel resistance in ovarian cancer. Owing to the central role of ubiquitination in the life activity of cells, the discovery of UBC13 function and the signaling pathway involved during the paclitaxel resistance process would accelerate the progress of studies on reversing paclitaxel resistance in ovarian cancer.

Here, we initially verified the effect of UBC13 in regulating the sensitivity of ovarian cancer cells and tissues to paclitaxel, based on DIGE quantitative proteomics. We further found, for the first time according to our knowledge, that paclitaxel-induced UBC13 down-regulation led to DNMT1 (DNA methyltransferase 1) degradation depression by decreased ubiquitination, which then resulted in reduced expression of CHFR (checkpoint with forkhead and ring finger domains) by promoter hypermethylation, and this consequently induced Aurora A (aurora kinase A) overexpression. This pathway may be a key mechanism by which ovarian cancer cells obtain the secondary resistance to paclitaxel, and UBC13 could potentially become a molecular drug in ovarian cancer therapeutics.

## Results

### Differential expression of proteins between paclitaxel-sensitive and paclitaxel-resistant ovarian cancer cells by proteomic analysis

**Fig. 1:**
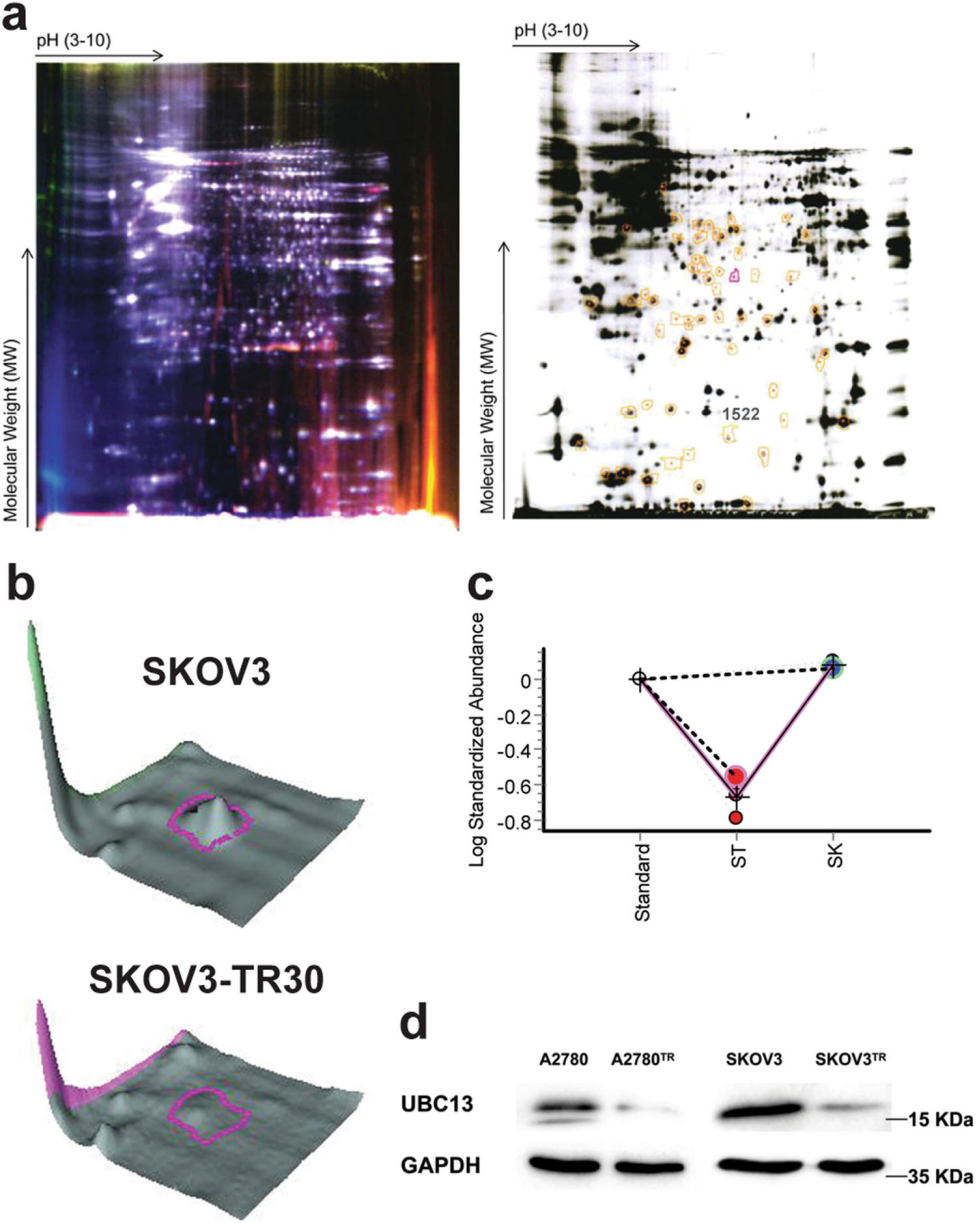
Differential expressions of proteins between paclitaxel sensitive and resistant ovarian cancer cells by proteomic analysis **a** Analysis of SKOV3 and SKOV3-TR30 samples by DIGE. Fluorescent (left) and monochrome (right) images of the DIGE Gel. Proteins extracted from SKOV3 (SK) and SKOV3-TR30 (ST) were labeled with Cy3 and Cy5, respectively. The labeled samples were initially separated in the first dimension (*x*-axis) on a non-linear gradient pH 3-10, then separated in the second dimension (*y*-axis) on a 12% polyacrylamide gel. The circled spots were identified by MALDI-TOF/TOF MS/MS analysis shown in the supplementary material Table S1–S3. **b** Three-dimensional volumetric models of UBC13 in SKOV3 (top) and SKOV3-TR30 (bottom) for DIGE spots. **c** The expression levels of UBC13 shown on DIGE were calculated by DeCyder analysis and presented as standardized log abundance. SK presents SKOV3 and ST presents SKOV3-TR30. **d** Western blotting of UBC13 in A2780 vs. A2780-TR and SKOV3 vs. SKOV3-TR30 cells, respectively. One of three representative results is shown in (**d**)

Paclitaxel-sensitive SKOV3 and paclitaxel-resistant SKOV3-TR30 cells served as our study models. In total, 57 protein spots were detected, and 49 of them were identified by DeCyder analysis (Supplementary Table S1). Figure 1a showed the superimposed image in pseudocolor from Cy3-labeled and Cy5-labeled samples combined with the monochrome image of the DIGE gel. We identified 60 proteins that were differentially expressed between SKOV3 and SKOV3-TR30 cells from 49 protein spots by the MALDI-TOF/TOF MS and MS/MS methods through IPI database, among which there were 38 down-regulated and 22 up-regulated proteins with more than 1.5-fold quantitative alterations in the SKOV3-TR30 cells. Of those, 20 were down-regulated by more than two-fold (maximum 9.42-fold) and 7 were up-regulated by more than two-fold (maximum 7.93-fold) in the SKOV3-TR30 cells (Supplementary Tables S2 and S3).

Next, in UBC13, one down-regulated protein with 5.58-fold quantitative alterations in paclitaxel-resistant cells by DIGE, was selected. Figure 1b showed a 3D volumetric model of UBC13 in SKOV3 and SKOV3-TR30 cells. Protein spot quantification of UBC13, performed by differential in-gel analysis, showed that SKOV3-TR30 cells had a lower UBC13 protein expression level than SKOV3 cells (Fig. 1c). Moreover, UBC13 expression was decreased in both paclitaxel-resistant SKOV3-TR30 and A2780-TR cells compared with their parental SKOV3 and A2780 cells (Fig. 1d), validated by western blot.

### The low-expression of UBC13 protein in ovarian cancer tissues is correlated with a poorer prognosis for patients

**Fig. 2:**
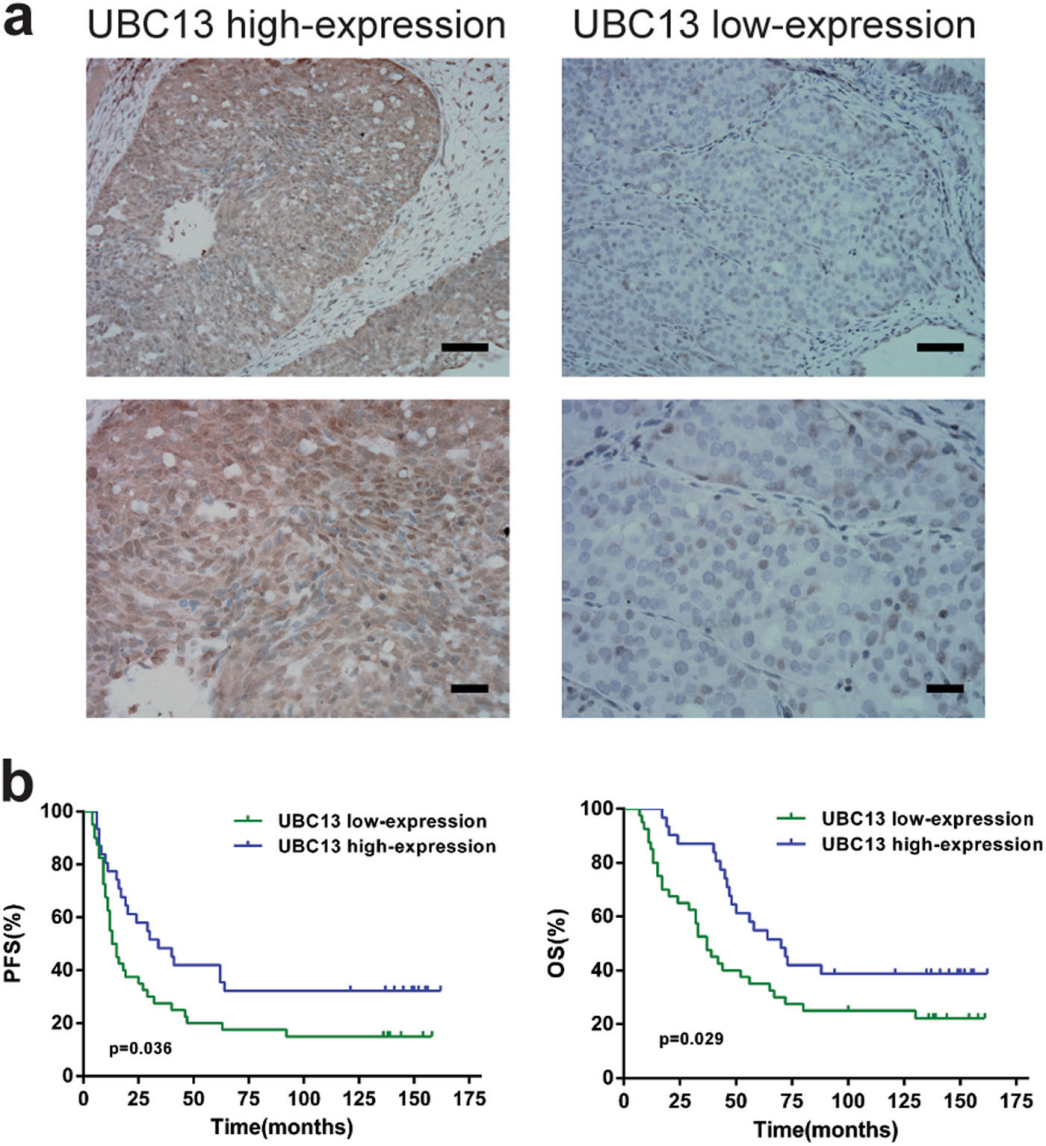
Analysis of the association of immunohistochemistry of UBC13 expression with the survival of patients **a** Representative UBC13 staining from ovarian cancer tissues. Scale bars represent approximately 500 μm (top) and 200 μm (bottom). **b** Kaplan-Meier survival curves for PFS and OS in ovarian cancer patients with low-expression and high-expression UBC13 protein by log-rank test

To further validate decreased UBC13 expression in paclitaxel-resistant ovarian cancer cells and understand its clinical significance, we detected the expression of UBC13 protein in 71 ovarian cancer tissues by immunohistochemistry (IHC). As expected, the ratio of UBC13 low-expression accounted for 75.75% (28/37) in the chemoresistant ovarian cancer tissues, and in contrast, only 35.3% (12/34) was present in chemosensitive tissues (Fig. 2a; Table 1), with a significant difference (*P* = 0.001). Furthermore, UBC13 low-expression was significantly correlated with a high-grade tumor (*P* = 0.038), larger volume of ascitic fluid (*P* = 0.006), suboptimal primary surgery (*P* = 0.023), and chemoresistance (*P* = 0.001) (Table 1). Kaplan-Meier survival curves revealed that ovarian cancer patients with UBC13 low-expression had significantly poorer progression-free survival (PFS, *P* = 0.036) and overall survival (OS, *P* = 0.029) than those with high UBC13 expression (Fig. 2b). Our results suggest that decreased UBC13 expression predicts a poorer prognosis for ovarian cancer patients and there may be a link between UBC13 expression and paclitaxel sensitivity in ovarian cancer cells.

**Table 1:** The relationship between UBC13 expression and clinicopathological parameters

Variable	*N*	UBC13 expression		*P* value
		Low	High	
Age (years)				0.156
>50	34	16	18	
≤50	37	24	13	
FIGO stage				0.076
I/II	15	5	10	
III/IV	56	35	21	
Tumor grade				0.038^*^
low-grade	22	8	14	
High-grade	49	32	17	
Ascitic fluid volume (ml)				0.006^**^
<500	44	19	25	
≥500	27	21	6	
Primary surgery				0.023^*^
Optimal	46	21	25	
Suboptimal	25	19	6	
Serum CA125 (U/ml)				0.053
<500	31	13	18	
≥500	40	27	13	
Chemosensitivity				0.001^**^
Sensitive	34	12	22	
resistant	37	28	9	

*P < 0.05, **P < 0.01

### Paclitaxel induces UBC13 down-regulation, and UBC13 modulates the paclitaxel sensitivity through the DNMT1, CHFR, and Aurora A pathway

**Fig. 3:**
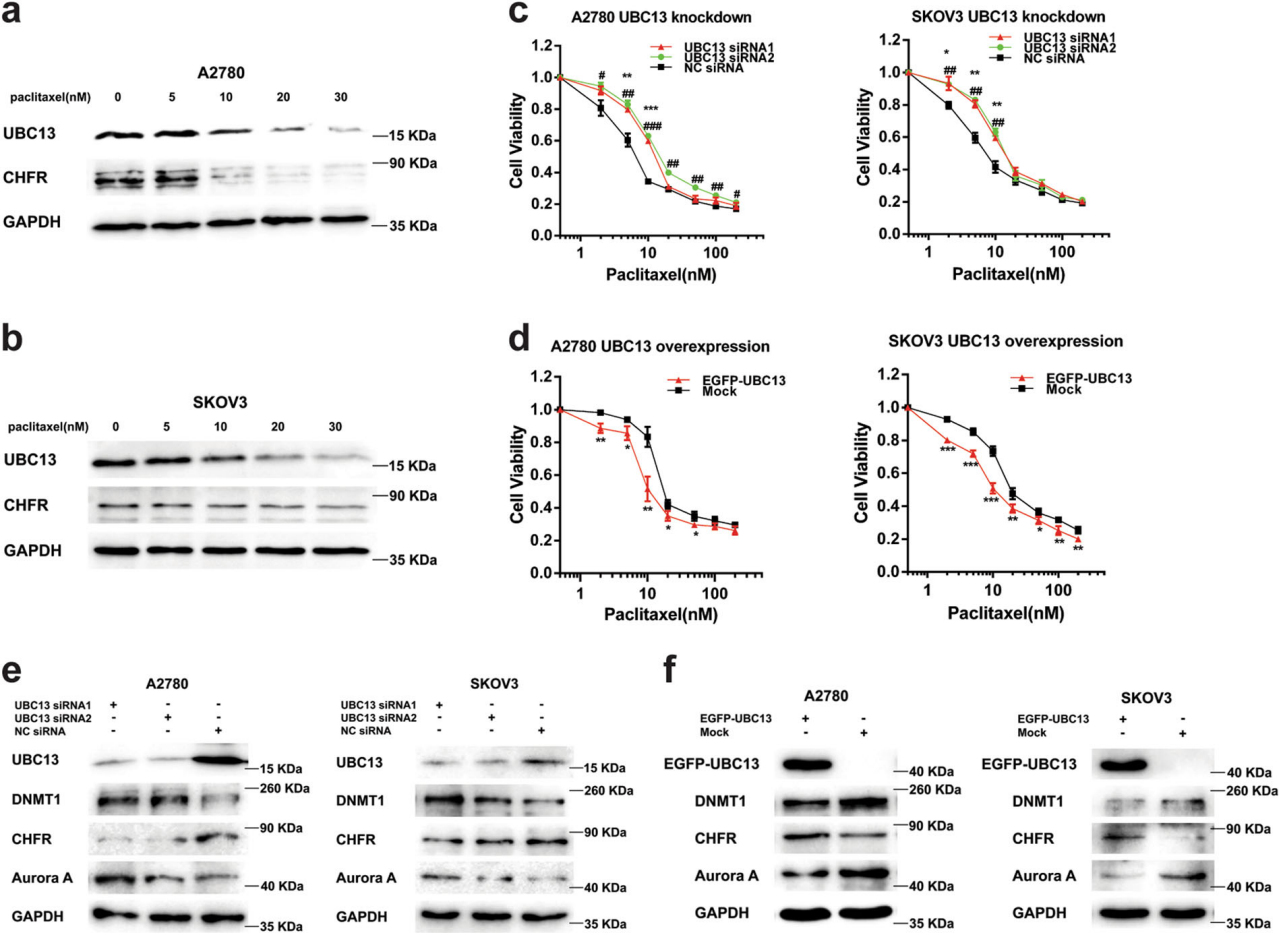
Paclitaxel induces UBC13 down-regulation, and UBC13 modulates the paclitaxel sensitivity through the DNMT1, CHFR, and Aurora A pathway **a** A2780 and **b** SKOV3 cells were treated with 5, 10, 20, and 30 nM paclitaxel for 24 h. Western blotting was performed with the indicated antibodies. Cell viability assays in A2780 and SKOV3 cells with **c** UBC13-knockdown and **d** UBC13-overexpression that were treated with paclitaxel at the indicated concentrations. Results are shown as means ± SEM for at least three separate experiments (* *P* < 0.05, ** *P* < 0.01, *** *P* < 0.001 for siRNA1 or plasmid; ^#^ *P* < 0.05, ^##^ *P* < 0.01, ^###^ *P* < 0.001 for siRNA2). Western blotting of UBC13, DNMT1, CHFR, and Aurora A in A2780 and SKOV3 cells with **e** UBC13-knockdown and **f** UBC13-overexpression. One of three representative results is shown in (**a**, **b**, **e**, and **f**)

A2780 and SKOV3 cells were exposed to paclitaxel at different concentrations (5, 10, 20, and 30 nM) for 24 h. The amount of UBC13 protein was decreased in both types of cells at the 20 and 30 nM paclitaxel exposure (Fig. 3a, b). Since paclitaxel is a cell cycle inhibitor, and CHFR, as a ubiquitin ligase (E3), is regarded as involved in a checkpoint regulating entry to mitosis and to arrest cancer cells at the G_2_-M phase after treatment with microtubule inhibitors, like paclitaxel^24,25^, we checked CHFR as a candidate for UBC13’s downstream modulator, and found that CHFR expression was synchronously decreased with UBC13 decline in both types of cells upon exposure to paclitaxel for 24 h (Fig. 3a, b). Then, we assessed the effects of altered UBC13 expression on paclitaxel sensitivity in A2780 and SKOV3 cells, and found that UBC13 knockdown by specific siRNA significantly protected ovarian cancer cells from paclitaxel (Fig. 3c). Conversely, UBC13 overexpression by transfection with a pEGFP-UBC13 plasmid significantly increased the sensitivity to paclitaxel in both types of cells (Fig. 3d). Simultaneously, CHFR expression was decreased and increased, respectively, following UBC13 down-regulation produced by UBC13 siRNA and up-regulation induced by UBC13 plasmid (Fig. 3e, f). It has been shown that CHFR could be regulated through its promoter methylation induced by DNA methyltransferases (DNMTs)^26^, except for a ubiquitination pathway. We tested the expression of DNMT1, one of the members of DNMTs, during the regulation of UBC13, and found that DNMT1 was increased when UBC13 was knocked down and decreased when UBC13 was over-expressed in A2780 and SKOV3 cells (Fig. 3e, f). We further found that Aurora A, which is negatively regulated by CHFR and involved in regulating the sensitivity of cancer cells to paclitaxel^25,27,28^, was synchronously increased following UBC13 knock-down and decreased with UBC13 up-regulation in both cells (Fig. 3e, f). Thus, our findings suggest that paclitaxel induces UBC13 down-regulation, which facilitates cell resistance to paclitaxel, and a pathway consisting of DNMT1, CHFR, and Aurora A probably participates in UBC13 regulation of the sensitivity to paclitaxel in ovarian cancer cells.

### UBC13 controls DNMT1 stability via ubiquitination and DNMT1 participates in UBC13 regulation of paclitaxel sensitivity

**Fig. 4:**
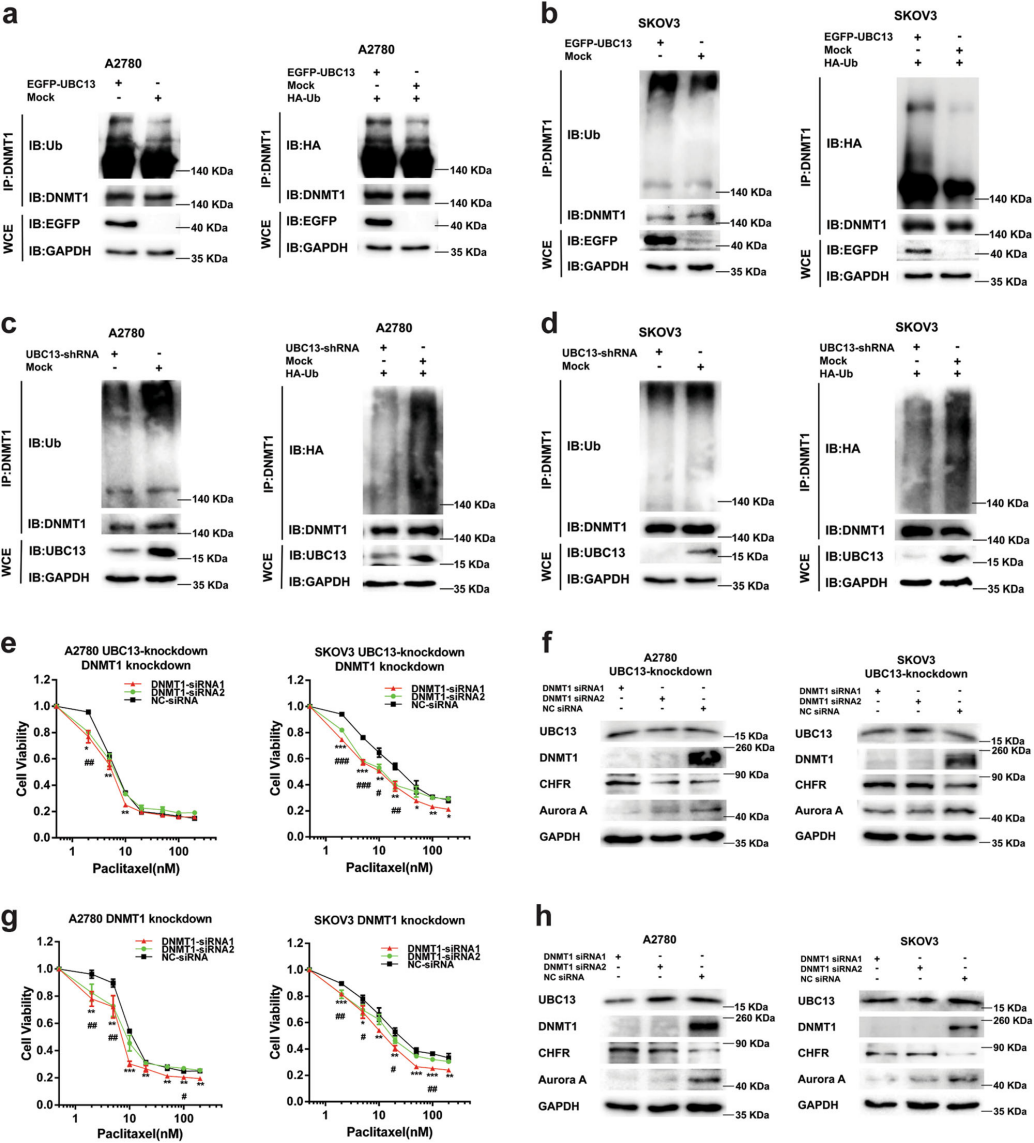
UBC13 controls DNMT1 stability via ubiquitination and DNMT1 participates in UBC13 regulation of the paclitaxel sensitivity DNMT1 ubiquitination in **a** A2780 and **b** SKOV3 cells with UBC13-overexpression or **c** A2780 and **d** SKOV3 with UBC13-knockdown without or with HA-ubiquitin. Cells were treated with MG-132 (20 μM, 8 h) prior to preparation of lysates and then subjected to IP followed by western blot with the indicated antibodies. **e** Cell viability assays in A2780 and SKOV3 cells with DNMT1-knockdown, which were transfected in advance with UBC13-specific shRNA and selected with G418 (400 μg/mL) for 14 days, and treated with paclitaxel at the indicated concentrations. **f** Western blotting of UBC13, DNMT1, CHFR, and Aurora A in A2780 and SKOV3 cells with DNMT1-knockdown, which were transfected in advance with UBC13-specific shRNA and selected with G418 (400 μg/mL) for 14 days. **g** Cell viability assays in A2780 and SKOV3 cells with DNMT1-knockdown, which were treated with paclitaxel at the indicated concentrations. **h** Western blotting of UBC13, DNMT1, CHFR, and Aurora A in A2780 and SKOV3 cells with DNMT1-knockdown. Results are shown as means ± SEM for at least three separate experiments in (**e** and **g**) (*P < 0.05, **P < 0.01, ***P < 0.001 for siRNA1; ^#^ *P* < 0.05, ^##^ *P* < 0.01, ^###^ *P* < 0.001 for siRNA2). One of three representative results is shown in (**a**–**d**, **f**, and **h**)

Since UBC13 is a ubiquitin-conjugating enzyme, we determined if UBC13 regulated DNMT1 through ubiquitination and found that DNMT1 ubiquitination was increased in A2780 and SKOV3 cells with UBC13 over-expression (Fig. 4a, b), whereas it was decreased in both cells with UBC13 knockdown (Fig. 4c, d). Meanwhile, the same results were observed when exogenous ubiquitin was co-expressed with UBC13-shRNA or pEGFP-UBC13 plasmid in A2780 and SKOV3 cells (Fig. 4a–d). Furthermore, we found that the DNMT1 protein half-life in the paclitaxel-resistant ovarian cancer cells was increased compared to the sensitive parental cells (Supplementary Figure S2a and b).

Then, we examined the influence of DNMT1 when UBC13 regulated the sensitivity to paclitaxel. After A2780 and SKOV3 cells with UBC13 knockdown were selected by G418 for 14 days, siRNAs specific to DNMT1 were transfected into both cells. The cytoprotection to the drug owing to UBC13 down-regulation was partially reversed by DNMT1 knockdown (Fig. 4e). Meanwhile, decreased CHFR expression and increased Aurora A expression induced by UBC13 knockdown were also partially reversed by DNMT1 reduction (Fig. 4f). In addition, DNMT1 knockdown by specific siRNA enhanced the sensitivity to paclitaxel (Fig. 4g), as well as increased CHFR and reduced Aurora A, but not UBC13, in both A2780 and SKOV3 cells (Fig. 4h). Our findings together suggest that UBC13 controls DNMT1 stability via ubiquitination and DNMT1, probably through CHFR and Aurora A, participates in UBC13 regulation of the sensitivity of ovarian cancer cells to paclitaxel.

### DNMT1 maintains CHFR gene expression via promoter methylation and CHFR participates in UBC13 regulation of paclitaxel sensitivity

**Fig. 5:**
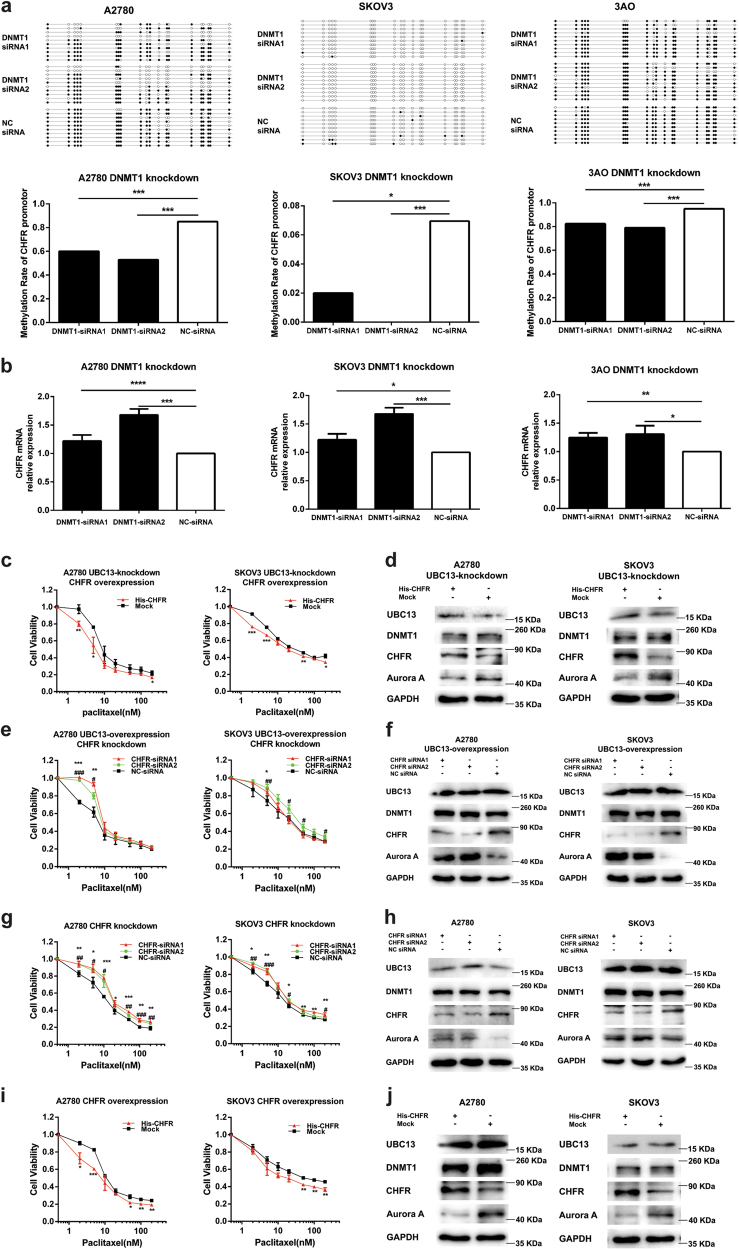
DNMT1 maintains CHFR gene expression via promoter DNA methylation, and CHFR participates in UBC13 regulating paclitaxel sensitivity **a** Detection of methylation status at the promoter region of the CHFR gene in A2780, SKOV3, and 3AO cells with DNMT1-knockdown by Bisulfite sequencing. Ten lines with circles represent the same sequence of ten clones from one sample. CpG sites are shown as filled circles (methylated) or unfilled circles (unmethylated). The lower panel shows the summary data. *P < 0.05, ***P < 0.001, compared with NC siRNA group (chi-square test). **b** mRNA expression of CHFR in A2780, SKOV3, and 3AO cells with DNMT1-knockdown. **c** Cell viability assays in CHFR-overexpression A2780 and SKOV3 cells, which were transfected in advance with UBC13-specific shRNA and selected with G418 (400 μg/mL) for 14 days, and treated with paclitaxel at the indicated concentrations. **d** Western blotting of the indicated proteins with CHFR overexpression, which were transfected in advance with UBC13 specific shRNA and selected with G418 (400 μg/mL) for 14 days. **e** Cell viability assays in A2780 and SKOV3 cells with CHFR-knockdown, which were transfected in advance with pEGFP-UBC13 plasmid and selected with G418 (400 μg/mL) for 14 days, and treated with paclitaxel at indicated concentrations. **f** Western blotting of the indicated proteins with CHFR knockdown, which were transfected in advance with pEGFP-UBC13 plasmid and selected with G418 (400 μg/mL) for 14 days. **g** Cell viability assays in A2780 and SKOV3 cells with CHFR-knockdown treated with paclitaxel at the indicated concentrations. **h** Western blotting of the indicated proteins with CHFR-knockdown. **i** Cell viability assays in A2780 and SKOV3 cells with CHFR-overexpression, which were treated with paclitaxel at the indicated concentrations. **j** Western blotting of UBC13, DNMT1, CHFR, and Aurora A in A2780 and SKOV3 cells with CHFR-overexpression. Results are shown as means ± SEM for at least three separate experiments in (**b**, **c**, **e**, **g**, and **i**) (*P < 0.05, **P < 0.01, ***P < 0.001, ****P < 0.0001 in (**a**, **c**, and **i**); *P < 0.05, **P < 0.01, ***P < 0.001 for siRNA1, ^#^ *P* < 0.05, ^##^ *P* < 0.01, ^###^ *P* < 0.001 for siRNA2 in (**e** and **g**). One of three representative results is shown in (**d**, **f**, **h**, and **j**)

Since DNMT1 is one of the DNA methyltransferases, we knocked down DNMT1 by specific siRNAs and observed whether genetic reduction of DNMT1 altered the methylation of the CHFR promoter and the expression of CHFR in ovarian cancer cells. As shown in Fig. 5b, the expression level of CHFR mRNA was increased when DNMT1 was knocked down in A2780, SKOV3, and 3AO cells. Consistent with the change in mRNA expression, bisulfite sequencing PCR (BSP) revealed a significant loss of 5-methylcytosine from the CHFR promoter CpG island in A2780 and 3AO cells, but did not in SKOV3 cells, which is probably attributed to a very low original level of CHFR methylation in SKOV3 cells (Fig. 5a). Moreover, the paclitaxel resistant ovarian cancer cells (A2780-TR and SKOV3-TR30) presented higher CHFR promoter methylation status (Supplementary Figure S3a and b) and lower CHFR mRNA levels (Supplementary Figure S3c) than sensitive parental cells. Our findings suggest that DNMT1 modulates CHFR expression by altering promoter methylation.

Then, we examined CHFR influence on the paclitaxel sensitivity of ovarian cancer cells. After A2780 and SKOV3 cells with UBC13 knockdown were selected by G418 for 14 days, a pcDNA3.1(+)- CHFR plasmid was transfected into the cells. The cytoprotection, as well as up-regulated Aurora A, due to UBC13 knockdown was partially reversed by CHFR overexpression (Fig. 5c, d). In contrast, When both cells with UBC13 overexpression were selected with G418 for 14 days, the cytotoxicity and down-regulated Aurora A owing to UBC13 overexpression were also partially reversed by CHFR knockdown; but neither UBC13 nor DNMT1 expressions were affected (Fig. 5e, f). Furthermore, down-regulated CHFR inhibited paclitaxel sensitivity, elevated Aurora A expression, but did not affect DNMT1 and UBC13 in ovarian cancer cells (Fig. 5g, h), and vice versa (Fig. 5i, j). Our findings suggest that CHFR via Aurora A participates in UBC13 regulation of paclitaxel sensitivity in ovarian cancer cells.

### The pathway consisting of DNMT1, CHFR, and Aurora A participates in UBC13 regulation of paclitaxel sensitivity in ovarian cancer cells

**Fig. 6:**
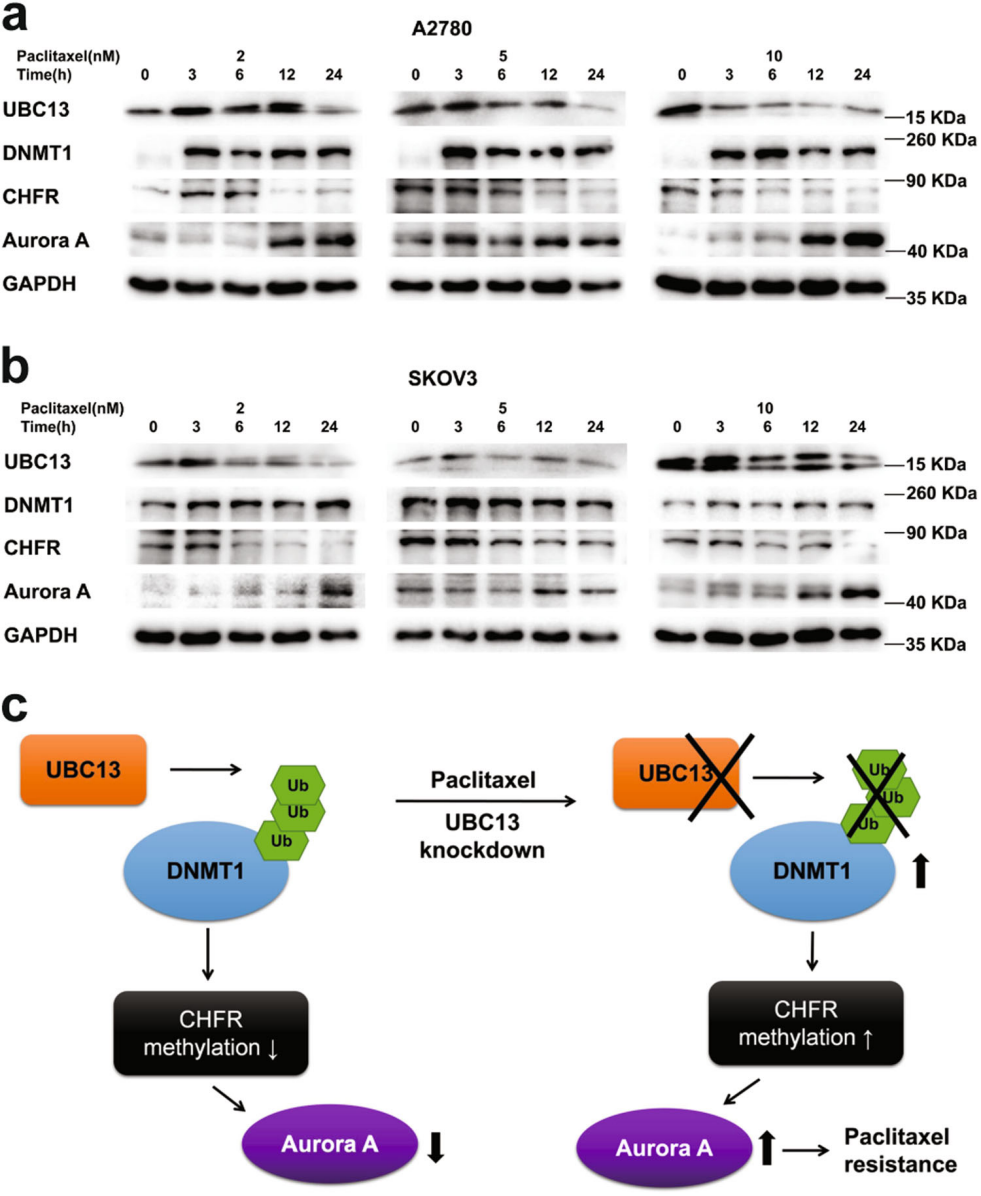
Paclitaxel exposure reduces UBC13 and CHFR, increases DNMT1 and Aurora A Western blotting of the indicated proteins in **a** A2780 and **b** SKOV3 cells treated with 2, 5, and 10 nM paclitaxel for the indicated times. Cells without paclitaxel treatment were assigned to be the blank control (0 h). One of three representative results is shown. **c** Working model: UBC13 regulates paclitaxel resistance via DNMT1/CHFR/Aurora A in ovarian cancer cells. UBC13 reduces DNMT1 through ubiquitination, results in DNA hypomethylation of the CHFR promoter, and decreases Aurora A. Reduction of UBC13 increases stability of DNMT1, leads to hypermethylation of the CHFR promoter, elevates Aurora A levels, and prompts paclitaxel resistance in ovarian cancer cells. Ub: ubiquitin

We cultured A2780 and SKOV3 cells with paclitaxel at different times (3, 6, 12, 24 h) and concentrations (2, 5, 10 nM), to observe the expression of the UBC13 protein, and the coinstantaneous response of the above four molecules in ovarian cancer cells to drug exposure. We found that the amount of UBC13 protein declined following the rise in drug exposure time or concentration in both cells, except for 2 nM at 12 h and earlier in the A2780 cells. Simultaneously, we observed that DNMT1 increased, CHFR decreased, and Aurora A increased following the UBC13 decline in both types of cells (Fig. 6a, b).

## Discussion

Chemoresistance is still one of the most important causations for treatment failure and death in ovarian cancer patients. Paclitaxel, as a microtubule inhibitor, is widely used in the therapy of ovarian cancer, as well as other solid tumors. However, the key mechanism related to its chemoresistance in ovarian cancer still remains to be clarified. Quantitative proteomic analysis technology provides an available approach to identify proteins involving chemotherapeutics resistance and to thoroughly understand the complicated mechanisms in cancers. Previously, high of numbers of differentially expressed proteins for chemoresistance have been identified by different proteomic techniques in ovarian cancer^29–31^. In this study, we identified 60 proteins differentially expressed at 1.5-fold or higher different intensities between SKOV3 and paclitaxel-resistant SKOV3-TR30 cell lines utilizing DIGE proteomic analysis combined with MS/MS Spectrometry. Of the 38 down-regulated proteins in SKOV3-TR30 cells, UBC13 was down-regulated with 5.58-fold different intensity and the difference was further verified in paired A2780-TR vs. A2780 and SKOV3-TR30 vs. SKOV3 cells by western blot. Thus, UBC13 is possibly involved in the paclitaxel resistance of ovarian cancer cells.

As a ubiquitin conjugating enzyme (E2), UBC13 plays a pivotal role in diverse biological processes such as protein degradation, the cell cycle, and DNA repair^6,19,32^. UBC13 also correlates with the development of some diseases^33–37^, and may even participate in regulating the chemotherapeutic sensitivity of tumors^23,38^. To validate the role of UBC13 in ovarian cancer chemoresistance, we detected the expression of UBC13 in 71 tissues of epithelial ovarian carcinoma by immunohistochemical staining, and found that the expression of UBC13 was significantly decreased in chemoresistant tissues compared with chemosensitive ones. Furthermore, clinicopathological analysis revealed that low-expression of UBC13 was significantly correlated to poorer prognostic factors like the high-grade of the tumor, larger volume of ascitic fluid, and suboptimal primary surgery. Additionally, patients with UBC13 low-expression showed shorter PFS and OS than those with UBC13 high-expression. This is the first report, as far as we know, on the reduced expression of UBC13 protein levels in paclitaxel-resistant cancer tissues and its clinical significance. To confirm the results in cells and tissues, we further examined the influence of paclitaxel on UBC13 expression and found reduced expression of UBC13 protein when ovarian cancer cells were exposed to paclitaxel at different concentrations. Inversely, the sensitivity of ovarian cancer cells to paclitaxel was decreased or increased when UBC13 was knocked down by siRNA or overexpressed by a pEGFP-UBC13 plasmid. Our results suggest that UBC13 may be a regulator of paclitaxel sensitivity in ovarian cancer cells.

As we know, UBC13 is an E2, and paclitaxel controls the cell cycle. Thus, the intersection of ubiquitination and the cell cycle pathway may be a position from which we can search for the downstream molecules of UBC13, by which UBC13 regulates paclitaxel sensitivity. CHFR is such a candidate because it is a ubiquitin ligase and acts as a checkpoint that delays the entry into metaphase in response to mitotic stress or microtubule inhibitors^24^. As we expected, we found that the CHFR expression was changed when the regulation of UBC13 expression altered the cellular sensitivity to paclitaxel. Moreover, CHFR decrease was consistent with UBC13 decline when ovarian cancer cells were exposed to paclitaxel. It is well known that CHFR transfers activated E2-conjugated ubiquitin to target proteins for degradation or other non-degradation progresses in the process of ubiquitination. Previous studies have also shown that CHFR is a RING-finger containing ubiquitin ligase whose level can be regulated by auto-ubiquitination^39,40^. However, in our study, CHFR expression was not depressed through auto-ubiquitination, while it presented synchronous up-regulation or down-regulation with increased or decreased UBC13 expression during either paclitaxel exposure or UBC13 regulation. Our results imply there may be another modulator between UBC13 and CHFR that neutralized CHFR auto-ubiquitination.

It has been reported that CHFR expression is also regulated by DNA methylation^26,41,42^. The low-expression of CHFR was concomitant with its promoter hypermethylation in lung and gastrointestinal cancers^26,41,43^. DNMT1, as one of the members of DNMTs, is mainly related to the precise duplicating and maintaining of DNA methylation. Moreover, the regulation of DNMT1 is correlated with ubiquitination-mediated degradation^44–46^. Thus, we supposed that UBC13 could regulate CHFR through DNMT1. As we expected, we found that DNMT1 expression was down-regulated with enhanced ubiquitination when UBC13 was overexpressed by a pEGFP-UBC13 plasmid, and contrarily, was up-regulated with weakened ubiquitination when UBC13 was knocked down by specific shRNA, confirming that UBC13 regulates DNMT1 through ubiquitination. Previous studies have shown that DNMT1 expression is controlled by acetylation. The increase of acetyltransferase Tip60 (Tat interactive protein 60 kDa) leads to DNMT1 acetylation, which, in turn, enhances its affinity with UHRF1 (ubiquitin like with PHD and ring finger domains 1) and activates DNMT1 ubiquitination and degradation^47^. Conversely, HDAC1 (histone deacetylase 1) induced DNMT1 deacetylation and HAUSP (herpes virus-associated ubiquitin-specific protease) modulated DNMT1 deubiquitination to stabilize DNMT1^44^. In this study, we report for the first time, to the best of our knowledge, that DNMT1 expression is regulated by UBC13 through ubiquitination. Furthermore, we found a reverse regulation of DNMT1 by siRNA in the resistance to paclitaxel induced by UBC13 down-regulation in ovarian cancer cells and a prolonged DNMT1 protein half-life in the paclitaxel resistant ovarian cancer cells, suggesting that DNMT1 participates in the process of UBC13 regulation of the sensitivity to paclitaxel in ovarian cancer cells.

DNA methylation contributes to many critical biological processes such as differentiation, X-chromosome inactivation, and DNA repair^48^, and it also correlates with the development and progression of cancers^49,50^, as well as chemotherapeutic resistance^51,52^. Some studies have reported that CHFR expression is modulated by DNA methylation of its promoter, however, most of those studies are limited to detecting the CHFR DNA methylation levels in cancer tissues^42,53,54^. Toyota et al. reported that both DNMT1 and DNMT3B knockout could induce CHFR promoter hypomethylation, but not by either DNMT1 or DNMT3B knockout alone, in colon cancer cells^26^. Yet in our study, we found that DNMT1 knockdown alone induced CHFR promoter hypomethylation significantly in A2780 and 3AO cells, which, in turn, led to increased expression of CHFR mRNA and protein in ovarian cancer cell lines. Moreover, CHFR promoter methylation status was higher and the CHFR mRNA level was lower in paclitaxel-resistant ovarian cancer cells. Our results suggest that DNMT1 regulates CHFR expression by altering its promoter DNA methylation in ovarian cancer cells, but such a regulating effect may be variable among different cancers.

Previous studies showed that CHFR level was elevated when cancer cells were stimulated with microtubule inhibitors, including paclitaxel, resulting in arrest of mitosis and escape from apoptosis induced by drugs^24,43^. The mechanism of this chemosensitivity regulation may be associated with the CHFR-inhibited Plk1 and/or Aurora A pathway^27,55,56^. Yet, some other studies revealed that the amplification of Aurora A was correlated with paclitaxel resistance. For instance, Hata et al. showed that knockdown of Aurora A by siRNA strengthened the paclitaxel chemosensitivity in pancreatic cancer cells^57^. Anand et al. and Giovinazzi et al. reported that overexpressed or stabilized Aurora A override the mitotic spindle assembly checkpoint and induced resistance to paclitaxel in cervical and breast cancer cells^28,58^. In addition, several studies identified that the expression of Aurora A was negatively regulated by CHFR^27,59^. In the present study, we found that CHFR overexpression decreased the level of Aurora A, and elevated the sensitivity to paclitaxel; and conversely, CHFR reduction increased the level of Aurora A and reduced the sensitivity to paclitaxel in ovarian cancer cells. Moreover, a reverse regulation of CHFR partally eliminated the resistance to paclitaxel induced by UBC13 regulation in ovarian cancer cells. Thus, our findings suggest that CHFR acts as a positive modulator in the process of UBC13 regulating paclitaxel sensitivity in ovarian cancer cells.

In summary, reduced expression of UBC13 is associated with poorer prognostic factors and shorter survivals in ovarian cancer patients. In a model (Fig. 6c) we illustrate that the exposure to paclitaxel induces UBC13 down-regulation, a decline in UBC13 increases DNMT1 expression via ubiquitination inhibition, elevated DNMT1 reduces the CHFR level through promoter hypermethylation, and subsequently up-regulates the amount of Aurora A in ovarian cancer cells. Such a process in its entirety enhances the protection to the cytotoxicity of the drug, and ultimately induces the secondary resistance of ovarian cancer cells to paclitaxel. Our findings reveal a novel mechanism by which UBC13 regulates paclitaxel sensitivity through a DNMT1-CHFR-Aurora A pathway in ovarian cancer cells. UBC13 could potentially be used as a therapeutic molecular drug for reversing paclitaxel resistance in ovarian cancer patients.

## Materials and methods

### Cell culture

Human ovarian cancer cell line SKOV3 was purchased from the American Type Culture Collection. A2780 cell line was purchased from Sigma. 3AO cell line was acquired from the Women’s Hospital, School of Medicine, Zhejiang University^60^. A2780-TR was a gift from Dr. Ding Ma at Tongji Hospital, Tongji Medical College, Huazhong University of Science and Technology, P.R. China. SKOV3-TR30, the paclitaxel-resistant sub-line, was developed as mentioned before^12^. Cell lines were maintained in McCoys’5 A or RPMI-1640 with 10% fetal bovine serum at 37 °C and 5% CO_2_. The resistant cell lines were maintained in 10 nM paclitaxel as appropriate.

### Reagents and antibodies

Paclitaxel was purchased from Bristol-Myers Squibb Pharmaceutical Ltd. MG-132(#S2619) was purchased from Sigma. The antibodies used for Western blotting included anti-UBC13 (#ab25885, Abcam), anti-DNMT1 (#24206-1-AP, Proteintech), anti-CHFR (#6904, Cell Signaling Technology), anti-Aurora A (#12100, Cell Signaling Technology), anti-Ub (#3933, Cell Signaling Technology), anti-HA(#ab003, Lianke), anti-EGFP (#ab006, Lianke), anti-GAPDH (#Mab5465, Lianke), anti-mouse HRP (#7076, Cell Signaling Technology), and anti-rabbit HRP (#7074, Cell Signaling Technology). Antibody anti-DNMT1 (#ab13537, Abcam) was used for immunoprecipitation.

### DIGE and protein identification by MALDI-TOF/TOF and MS/MS

Protein sample preparation was based on the protocol described by Unlu et al^61^. Briefly, protein sample extracts (50 μg) were mixed with Cy2, Cy3, or Cy5 and combined with rehydration buffer and then were loaded on 18-cm 3–10 NL pH range IPGstrip overnight. Isoelectric focusing (IEF) was performed on an IPGphor unit at 20 °C with the following voltage program: 12 h at 30 V, 1 h at 500 V, 1 h at 1000 V, and 6 h at 8000 V. After IEF, the IPG strips were reduced in equilibration buffer and then incubated in the same buffer with 2.5% w/v iodoacetamide. The second dimension separation was performed on polyacrylamide gels.

Labeled proteins were then visualized by scanning and images were processed using DeCyder 2D V6.5 software (GE Healthcare) to acquire the average ratio, unpaired Student’s *t*-test^62^. Protein spots expressed differentially with statistical significance were considered as follows: (i) at least appeared in 70% of gel images, (ii) fold change threshold, ≥1.5, and (iii) *P*-values less than 0.05.

DIGE gels were then stained with Coomassie, and differentially expressed spots were excised from the gels. The proteins were processed and digested following a procedure elsewhere^63^. The samples were analyzed with a 4800 Plus MALDI TOF/TOF Analyzer (Applied Biosystems) and the eight most intense precursor ions from each MS spectra were then MS/MS analyzed with CID. Protein candidates combining peptide mass fingerprinting/tandem MS search were analyzed by the MASCOT version 2.2 (Matrix Science Ltd) search engine against the International Protein Index (IPI) human database (downloaded in Dec 10, 2010, 86,702 sequences). The threshold of expectation value utilized for protein identification in this study was based on the significance level suggested by MASCOT, which is an expectation value below 0.05 (*P*-value < 0.05).

### Patient-specimen selection and IHC

Seventy-one formalin-fixed and paraffin-embedded tissue samples obtained from patients diagnosed as ovarian serous and endometrioid adenocarcinomas from February 2002 to June 2009 were used for IHC analyzes. All the patients underwent primary surgery followed by paclitaxel-based chemotherapy. The deadline of follow-up was September 30, 2016. The standard for determining paclitaxel resistance or sensitivity and the count of PFS and OS were described previously^64^. All of the pathological diagnoses were reconfirmed by a pathologist. The project was approved by the Ethical Committee (Women’s Hospital, School of Medicine, Zhejiang University) and written informed consent was acquired from patients prior to treatment. Analysis of IHC was performed as previously described^30^. Anti-UBC13 antibody (1:500) was used for the IHC. The scoring details were described previously (Figure S1)^65^.

### Plasmids and siRNA transfection

Full-length UBC13 was cloned into the pEGFP-C1 vector, His-tagged CHFR and HA-tagged Ub were cloned into the pcDNA3.1(+) vector, UBC13-specific shRNA was cloned into the pGPH1/Neo vector. The sequence of UBC13-shRNA was the same as UBC13-siRNA1. X-treme GENE HP DNA Transfection Reagent (#06366236001, Roche) was used for transfection. For G418 (#G5013, Sigma) selection, cells were transfected with pEGFP-UBC13 plasmid or pGPH1-shUBC13 plasmid for 24 h and treated with 400 μg/mL G418 for 14 days.

The siRNAs against UBC13 (siRNA 1, 5′-GGCUAUAUGCCAUGAAUAA-3′; siRNA 2, 5′-CCAGAUGAUCCAUUAGCAA-3′), CHFR (siRNA 1, 5′-CUGUAGAAUUGUAGUGGAU-3′; siRNA 2, 5′-GUGCAAAGUAUGGAUGCCA-3′), DNMT1 (siRNA 1, 5′-CACTGGTTCTGCGCTGGGA-3′; siRNA 2, 5′-CAAUGAGACUGACAUCAAA-3′) were used. The siRNAs were transfected into cells with DharmaFECT Transfection Reagents (#T-2001, GE Healthcare).

### Western blotting

Western blotting was performed on whole-cell extracts by lysing cells in buffer as described previously^65^. Immunoblots were detected with an Immobilon Western HRP Substrate (#WBKLS0100, Merck Millipore) in Imagequant LAS 4000 mini (GE Healthcare).

### Cell viability assays

A2780 and SKOV3 cells were transfected with plasmid or siRNA for 24 h. Then an appropriate quantity of cells was seeded (4000 cells per well) in 96-well plates. A2780 and SKOV3 cells were then exposed to paclitaxel at various concentrations (0, 2, 5, 10, 20, 50, 100, and 200 nM) for 48 h after adhering to the plates. The cell viability was determined with CellTiter 96 AQueous One Solution Cell Proliferation Assay (#G3580, Promega).

### In vivo DNMT1 ubiquitination assay

For in vivo DNMT1 ubiquitination assays, pEGFP-UBC13 with or without HA-ubiquitin were transfected into A2780 and SKOV3 cells for 24 h, pGPH1-shUBC13 with or without HA-ubiquitin were transfected into both cells for 48 h. Then the cells were treated with 20 μM of MG-132 for 8 h and lysed in IP Lysis Buffer (#87787, Thermo Scientific), 20 μM MG-132, and a protease-inhibitor cocktail. The lysates were incubated with anti-DNMT1 antibody and protein G magnetic beads (#1614833, Bio-Rad) at 4 °C overnight. The proteins were released from the beads by boiling in SDS-PAGE loading buffer and analyzed by western blot with anti-Ub or anti-HA antibodies.

### BSP assay

The genomic DNA of DNMT1 siRNA-transfected A2780, SKOV3, and 3AO cells were extracted 48 h after transfection using the QIAamp DNA Mini Kit (#51304, QIAGEN). The 500 ng genomic DNA was treated with sodium bisulfite using EZ DNA Methylation-Gold Kit (#D5005, Zymo Research). The 100 ng bisulfite-treated DNA was used for PCR amplification. The specific primer (forward: 5′-TTTTATTTTTAGGGAATATTTTTTGG-3′ and reverse: 5′-TACACACAATAAATCACACAAATCC-3′) was used. PCRs were performed using ZymoTaq^TM^ Premix Kit (#E2004, Zymo Research). The amplified PCR products were cloned into pEASY-T5 Zero Cloning Vector (#CT501, Transgen Biotech), and individual clones were sequenced using M13 as primers. Ten clones of each sample were analyzed and the methylation rate of whole CpG sites for each sample was calculated as the sum methylation rate of each sample.

### Quantitative real-time PCR (qRT-PCR)

RNA extraction kit (#9767, TaKaRa) was used to extract RNA from cultured cells. The cDNA was generated using the PrimeScript RT reagent Kit (#RR047, TaKaRa). Evaluation of CHFR mRNA level was performed by using SYBR Premix Ex Taq Kits (#RR042A TaKaRa) analyzed by qRT-PCR. The primer sequences of CHFR (forward: 5′-GGCAACCAGAGGTTTGACAT-3′ and reverse: 5′-AGTCAGGACGGGATGTTACG-3′) and GAPDH (forward: 5′-GACAGTCAGCCGCATCTTCT-3′ and reverse: 5′-TTAAAAGCAGCCCTGGTGAC-3′) were used. The 2^−ΔΔCt^ method was used to evaluate the gene expression fold change among the groups. Three independent experiments were performed.

### Statistical analysis

All statistics were conducted with SPSS version 19.0 (IBM Corp, USA). The differences in proteomic analysis between SKOV3 and SKOV3-TR30 were estimated using the Student’s *t*-test. The correlations between the IHC of UBC13 and clinicopathological parameters were evaluated by chi-square tests. PFS and OS curves were determined using the Kaplan-Meier method and the differences in survival were compared by Log-rank test. Cell viability and CHFR mRNA expression levels between the different groups were evaluated by Student’s *t*-test. Methylation rates for the CHFR promoter between groups were evaluated by chi-square test. A *P*-value of less than 0.05 was regarded as significant.

## Electronic supplementary material

Supplementary information

Figure S1

Figure S2

Figure S3

Dataset S1

Dataset S2

Dataset S3
