# Supplementary material for: The inhibition of UBC13 expression and blockage of the DNMT1-CHFR-Aurora A pathway contribute to paclitaxel resistance in ovarian cancer
Source: Cell Death Dis. 2018 Jan 24;9(2):93. doi: 10.1038/s41419-017-0137-x (PMC5833742; doi:10.1038/s41419-017-0137-x)
Supplement: Supplementary file 1 — Supplementary information [file 41419_2017_137_MOESM1_ESM.docx]

**Supplementary information**

**Supplementary Figure S1.** The intensity of immunohistochemical staining of UBC13 in ovarian cancer tissues. Representative images of UBC13 staining in ovarian cancer tissues were shown. Scale bars represent approximately 500 μm (top) and 200 μm (bottom). The intensity of staining is graded as follows: 1+, weak; 2+, moderate; 3+, strong; 4+, very strong.

**Supplementary Figure S2.** The half-life of DNMT1 protein was increased in the paclitaxel-resistant ovarian cancer cell lines in comparison to the sensitive parental cell lines. (**a**) A2780 and A2780-TR cells and (**b**) SKOV3 and SKOV3-TR cells were treated with 50 μM CHX for 5, 10, 20, and 30hr. Western blotting performed with the indicated antibodies.

**Supplementary Figure S3.** The paclitaxel-resistant ovarian cancer cells had higher levels of the CHFR promoter DNA methylation and lower levels of the CHFR mRNA expression than the sensitive parental cells. (**a**) Detection of DNA methylation status at the promoter region of the CHFR gene in A2780, A2780-TR, SKOV3, and SKOV3-TR cells by Bisulfite sequencing. Ten lines with circles represent the same sequence of ten clones from one sample. CpG sites were shown as filled circles (methylated) or unfilled circles (unmethylated). (**b**) The statistical significance of DNA methylation levels of the CHFR promoter between paclitaxel-resistant cells and sensitive parental cells, ** *P* < 0.01, *** *P* < 0.001. (**c**) The mRNA expression of CHFR in A2780, A2780-TR, SKOV3, and SKOV3-TR cells, *** *P* < 0.001, **** *P* < 0.0001.

**Supplementary Table S1.** Protein spots analysis of DIGE proteomic in SKOV3 vs. SKOV3-TR30 cells. Provided as an Excel file.

**Supplementary Table S2.** All proteins identified by MALDI TOF/TOF MS and MS/MS of DIGE proteomic in SKOV3 vs. SKOV3-TR30 cells. Provided as an Excel file.

**Supplementary Table S3** Screened out proteins of DIGE proteomic in SKOV3 vs. SKOV3-TR30 cells. Provided as an Excel file.

**Supplementary experimental procedures**

**Protein half-life assay**

A2780, A2780-TR and SKOV3, SKOV3-TR30 cells were treated with 50 μM CHX for 5, 10, 20, and 30hr. Then cells were harvested and protein was extracted and detected for western blotting.
