# Supplementary figures and images for: The inhibition of UBC13 expression and blockage of the DNMT1-CHFR-Aurora A pathway contribute to paclitaxel resistance in ovarian cancer
Source: Cell Death Dis. 2018 Jan 24;9(2):93. doi: 10.1038/s41419-017-0137-x (PMC5833742; doi:10.1038/s41419-017-0137-x)

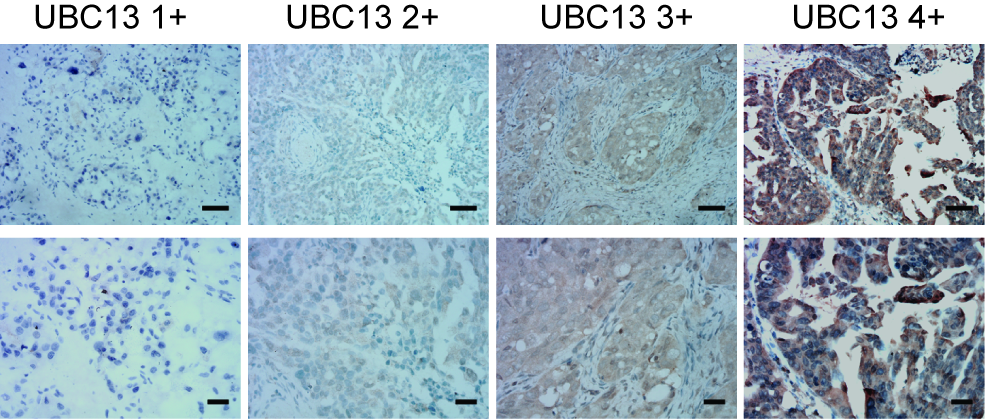

Supplement: Supplementary file 2 — Figure S1 [file 41419_2017_137_MOESM2_ESM.tif]

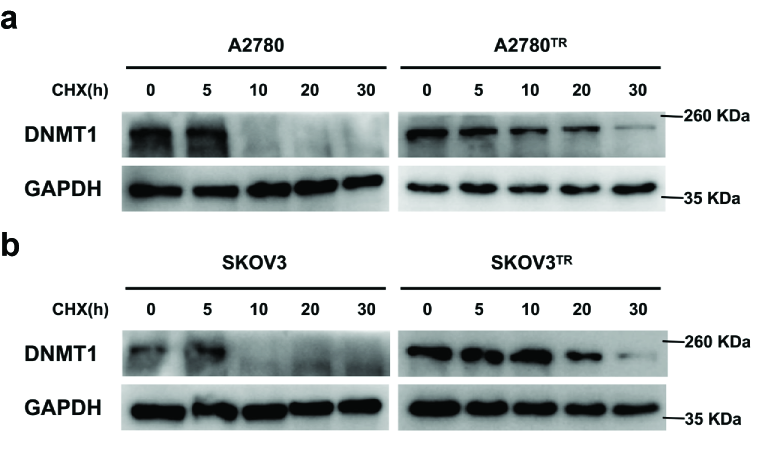

Supplement: Supplementary file 3 — Figure S2 [file 41419_2017_137_MOESM3_ESM.tif]

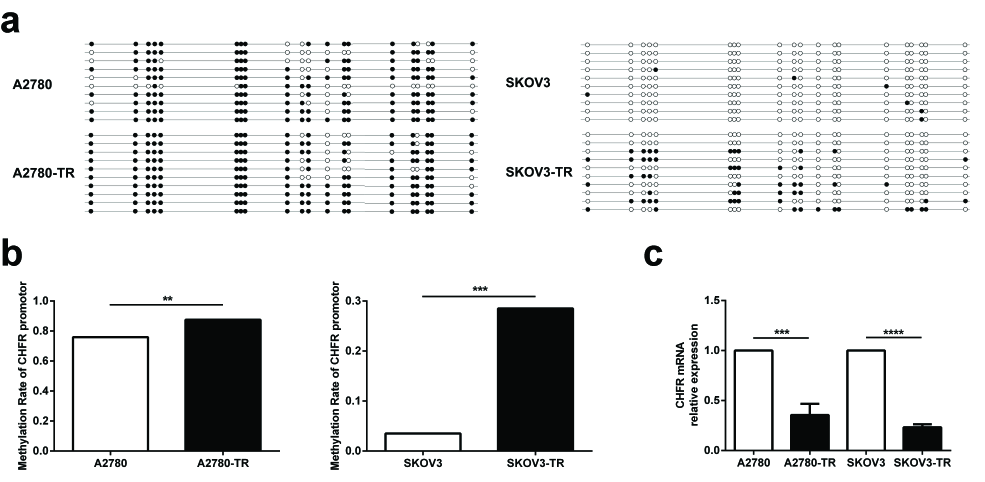

Supplement: Supplementary file 4 — Figure S3 [file 41419_2017_137_MOESM4_ESM.tif]
